# The impact of a multifaceted intervention on antibiotic use for common infections in nursing homes in Spain. A before and after study

**DOI:** 10.1007/s41999-025-01193-0

**Published:** 2025-04-22

**Authors:** Ramon Monfà, Ana García-Sangenís, Rosa Morros, Carlota Manuela Zárate Sáez, Jesús Mateos-Nozal, María N. Vaquero Pinto, Carmen Sáez Bejar, Elena López Pérez, Consuelo Rodríguez Jiménez, Rosa Magallón-Botaya, Priscila Matovelle, Alicia Navarro Sanmartín, Carl Llor

**Affiliations:** 1 Fundació Institut Universitari per a la Recerca a l’Atenció Primària de Salut Jordi Gol, Barcelona, Spain; 2 Universitat Autònoma de Barcelona, Bellaterra (Cerdanyola del Vallès), Spain; 3 CIBER en Enfermedades Infecciosas Instituto Carlos III, Madrid, Spain; 4 Department of Geriatrics, Hospital Universitario Ramón y Cajal (IRYCIS), Madrid, Spain; 5 Department of Internal Medicine, Hospital Universitario La Princesa, Instituto de Investigación Sanitaria (IIS-Princesa), Madrid, Spain; 6 Institut Català de la Salut, Barcelona, Spain; 7 Department of Pharmacology, Complejo Hospitalario Universitario de Canarias, Santa Cruz de Tenerife, Spain; 8 Group B21-23R, Health Research Institute of Aragon (IISA), Saragossa, Spain; 9 Network for Research on Chronicity, Primary Care, and Health Promotion (RICAPPS, Rd21/0016/0005), Saragossa, Spain; 10 Department of Primary Care, Universidad Zaragoza, Saragossa, Spain; 11 Department of Geriatrics, Hospital San Juan de Dios, Saragossa, Spain; 12 Nursing Home, Aragon Institute of Social Services (IASS), Borja, Spain; 13 Research Unit for General Practice, Department of Public Health, University of Southern Denmark, Odense, Denmark

**Keywords:** Nursing homes, Frail elderly, Hygiene, Antimicrobial stewardship, Medical audit, Overdiagnosis, Anti-bacterial agents

## Abstract

**Purpose:**

The aim of this study was to assess the impact of a multifaceted intervention aimed at nursing home staff on antibiotic use and hygiene elements for nursing home residents with common infections.

**Methods:**

Before-and-after study carried out in nursing homes in five areas of Spain. Nursing staff registered residents with common infections and documented hygiene practices over three months, before and after a 2-h educational intervention in autumn 2023. The intervention focused on the initial registration results, antibiotic guidelines, infection prevention, and management strategies for nursing homes. Indicators for potentially unnecessary antibiotic use and non-first-line antibiotics were developed, and results from both registration periods (Feb-Apr 2023 and Feb-Apr 2024) were compared to assess impact.

**Results:**

Of 34 nursing homes, 23 completed the intervention and the two registrations (67.6%). Staff reported 1003 infections in the first registration and 789 in the second. The overall antibiotic prescription rate was 84.6%. Potentially inappropriate antibiotic use for urinary tract infections decreased from 70.3 to 59.9% (*P* < 0.005) and use for respiratory infections dropped from 78.1 to 62.7% (*P* < 0.001), with inappropriate use decreasing from 46.3 to 31% (*P* < 0.001). The mean duration of antibiotics for cystitis reduced from 7 to 5.4 days (*P* < 0.05). The intervention had no impact on hygiene practices.

**Conclusion:**

Potentially unnecessary antibiotic use was lower after the intervention, whereas hygiene practices were unchanged. A more intensive, multifaceted educational approach is needed for a greater impact.

**Supplementary Information:**

The online version contains supplementary material available at 10.1007/s41999-025-01193-0.

## Key summary points

**Aim:**

Evaluate nursing staff intervention’s impact on antibiotic use and hygiene for nursing home residents’ infections.

**Findings:**

A 2-h intervention for nursing staff, involving individual and group feedback, evidence-based infection management, and infection prevention techniques, improved prescription appropriateness for common infections in nursing homes.

**Message:**

The intervention was effective, but a more comprehensive educational program is required.

**Supplementary Information:**

The online version contains supplementary material available at 10.1007/s41999-025-01193-0.

## Introduction

Life expectancy in affluent countries is rising, with Europe’s old-age dependency ratio projected to reach 50% by 2050 [1]. Nursing home residents are particularly susceptible to frequent and severe infections, with respiratory and urinary tract infections being especially common [2]. Older adults, particularly those in nursing homes, present a high prevalence of infections [3]. Although recent data are limited, an estimated 2-million infections occur annually in nursing homes in the United States [4].

Antibiotic overprescribing in nursing homes is high, with at least 60% being inappropriate, leading to multidrug-resistant uropathogens [5]. Watch and Reserve broad-spectrum antibiotics, as defined by the World Health Organization Access, Watch, Reserve (AWaRe) classification [6], are often used, worsening antimicrobial resistance and increasing morbidity, mortality, and societal costs [7]. Antibiotic prescribing is complex and influenced by social and organizational factors, with overdiagnosis contributing to overuse [8–11]. Resistant bacteria can spread through contact, making infection prevention crucial. Evidence-based antimicrobial stewardship and infection control policies are needed to promote appropriate antibiotic use and prevent infection spread in nursing homes [12, 13]. Our objective was to assess the impact of a multifaceted intervention aimed at nursing home staff on antibiotic use and hygiene elements for nursing home residents with common infections.

## Methods

### Setting, study population and study design

This study followed a before/after intervention approach with the implementation of a quality control methodology. In autumn 2022, a minimum of five nursing homes or wards from large nursing homes across five different regions in Spain were recruited. To assess the effectiveness of the intervention, we conducted two-point prevalence audits. These audits were performed before and after the intervention, with participating nursing staff professionals recording anonymized data on consultations related to common infections and hygiene elements using two different forms. Each nursing home recruited at least one nursing staff professional to collect data on infections in a form during each audit registration.

## Audit registrations

During the audit period, nursing staff professionals used a simple registration form to record all consultations regarding common infections. They filled in the form sequentially for each consultation related to the selected type of infection. The form consisted of 18 main groups of items, with 88 variables, organized in 2 sections. The first part collected general information about the infection (patient demographic data, type of infection, antibiotic treatment, allergies, perception of antibiotic demand and hospital referrals) whereas the second part gathered more information from urinary tract and respiratory infections (signs and symptoms, diagnosis and test used). Figure S1 displays the original version of the form that professionals were required to complete during the two registration periods. Each case was recorded on one line, with professionals typically only needing to tick boxes, avoiding written responses. A pilot test was conducted in January 2023 with two nursing staff professionals per region ensured that the registration form was relevant to their practice, easily understood, and confirmed that enough cases were available.

In addition to this registration form, professionals were asked to complete another form regarding hygiene elements available in the nursing home on the first day of the two registration periods. Two of the authors gathered hygiene elements from the medical literature, and prior to the start of the study, the entire consortium prioritized a list of 27 hygiene elements, covering different aspects of hygiene: hand hygiene, hygiene resources at the nursing home, protective equipment, specific hygiene resources, professional hygiene management, resident and family hygiene management, perineal care, and diaper changing.

## Intervention

All the professionals undertook a 2-h educational intervention in each of the five regions in autumn 2023. This intervention was either physical or online and included the following elements: (a) individual feedback and group discussion on the results of the first registration at both an individual and group level regarding the appropriateness of the diagnostic approach, antibiotic use, and type of antimicrobials used for the treatment of common infections and hygiene elements implemented, (b) evidence-based management of common infections based on updated national guidelines, including an explanation of the natural course, role of testing, and management, and (c) teaching and implementation of infection prevention and control measures in nursing homes. A report with the results of the first registration, as well as recommendations for respiratory and urinary tract infections, was distributed to each participant.

## Data analysis

We analyzed the antibiotic prescribing rates during the two registration periods. We developed two indicators to assess the impact of the intervention. The first indicator, potentially unnecessary antibiotic use, refers to an antibiotic prescribed to a patient with a suspected urinary or respiratory tract infection that did not meet the minimum criteria for prescribing an antibiotic, as defined by medical literature, which are summarized in Fig. S2. We primarily relied on international guidelines [14–17] for this, and for suspected urinary tract infections, we applied the algorithm proposed by van Buul in 2018 for both residents with and without indwelling urinary catheters [8]. The second indicator measured the percentage of first-line antibiotic use in accordance with national guidelines [18, 19].

We applied the Fisher exact test to the frequencies of antibiotic courses before and after the audit to test the null hypothesis of no effect of the intervention. Student t tests were used to compare quantitative variables. Statistical significance was considered at *P* < 0.05. The data were analyzed with the R v4.3 statistical program.

## Results

### Study population

A total of 34 nursing homes participated in the first registration period, with 23 (67.6%) undergoing the intervention and participating in the second registration period. As shown in Table S1, the demographic characteristics of the residents, types of infections, antibiotic prescribing rates, initiation of antibiotic courses, and referrals to the hospital were similar among the initial nursing homes and those that completed the intervention and second registration. The main results presented here are based on data from nursing home staff who participated in both registration periods (2023 and 2024). They reported 1003 infections during the first registration and 789 during the second registration. Table 1 provides a detailed summary of the main results.

**Table 1:** General characteristics of the residents from the nursing homes that completed the two registration audits

Characteristics	Total	First registration	Second registration	*P* value
Number of nursing homes	23	23	23	
Number of infections/registrations	1792	1003	789	
Age, year, mean (SD)	86.6 (8.2)	86.4 (8.2)	86.9 (8.1)	0.229
Female gender, *n* (%)	1306 (73.0)	711 (71.0)	595 (75.7)	0.029
Previous duration of symptoms, mean days (SD)	2.3 (3.6)	2.4 (4.2)	2.1 (2.7)	0.035
Type of infection, *n* (%)				
Urinary tract infection	772 (43.1)	475 (47.4)	297 (37.6)	< 0.001
Respiratory tract infection	762 (42.5)	392 (39.1)	370 (46.9)	
Skin and soft-tissue infection	144 (8.0)	79 (7.9)	65 (8.2)	
Other infections*	114 (6.4)	57 (5.7)	57 (7.2)	
Antibiotic prescribing rate, *n* (%)	1516 (84.6)	886 (88.3)	630 (79.8)	< 0.001
Indication for antibiotic therapy, *n* (%)				
Initiation of treatment	1415 (79.0)	819 (81.7)	596 (75.5)	0.002
Prophylaxis	49 (2.7)	37 (3.7)	12 (1.5)	0.008
Continuation	63 (3.5)	42 (4.2)	21 (2.7)	0.107
Unknown	10 (0.6)	3 (0.3)	7 (0.9)	0.180
Antibiotic taken during the previous 15 days, *n* (%)	1458 (81.4)	812 (81.0)	646 (81.9)	0.664
Where the antibiotic course was initiated *n* (%)				
Nursing home	1321 (73.7)	783 (78.1)	538 (68.2)	< 0.001
Hospital	181 (10.1)	94 (9.4)	87 (11.0)	0.282
Healthcare center	17 (0.9)	10 (1.0)	7 (0.9)	1.000
Unknown	6 (0.3)	1 (0.1)	5 (0.6)	0.126
Duration of the antibiotic course, day, mean (SD)	7.2 (9.8)	7.0 (7.5)	7.5 (12.3)	0.388
Allergy to penicillin, *n* (%)	47 (2.6)	22 (2.2)	25 (3.2)	0.257
Perception of demand for antibiotic**, *n* (%)	643 (35.9)	395 (39.4)	248 (31.4)	0.001
Referral to hospital, *n* (%)	241 (13.4)	128 (12.8)	113 (14.3)	0.373

SD = Standard deviation

*Infections other than respiratory, urinary, and skin and soft-tissue infections

**It includes the verbal request expressed by family members and/or the residents themselves, but also the perception of the nursing staff that they wanted an antibiotic regimen, even if it has not been verbally requested

## Urinary and respiratory infections

Urinary tract infections accounted for 772 cases across both registrations, while 762 cases were specifically identified as respiratory tract infections. Together, these two types of infections constituted 85.6% of the total infections registered. The mean age of the residents included in the forms was 86.6 years (standard deviation [SD] 8.2 years), with 73% being women (Table 1). The most frequently reported diagnosis in both registrations was cystitis, with 586 cases, followed by common cold and acute bronchitis, with 184 and 139 episodes, respectively (Table 2).

**Table 2:** Percentage of diagnoses, antibiotic prescription rate and mean duration for all infections listed in the registration form, before and after the intervention

Diagnosis	First registration			Second registration		
	Cases, *n* (%)	Antibiotics given, *n* (%)	Duration, days, mean (SD)	Cases, *n* (%)	Antibiotics given, *n* (%)	Duration, days, mean (SD)
Respiratory tract infections	392 (100.0)	306 (78.1)	6.7 (1.7)	370 (100.0)	232 (62.7)	6.8 (1.6)
Common cold	87 (22.2)	27 (31.0)	6.8 (1.7)	97 (26.2)	9 (9.3)	6.0 (2.4)
Acute otitis media	2 (0.5)	2 (100.0)	7.0 (0.0)	3 (0.8)	3 (100.0)	6.3 (1.1)
Acute sinusitis	0 (0.0)	–	–	0 (0.0)	–	–
Acute pharyngitis	22 (5.6)	20 (90.9)	5.8 (1.9)	17 (4.6)	7 (41.2)	6.1 (1.6)
Acute tonsillitis	8 (2.0)	8 (100.0)	6.7 (3.8)	2 (0.5)	2 (100.0)	7.0 (0.0)
Acute bronchitis	77 (19.6)	70 (90.9)	6.5 (1.2)	62 (16.8)	49 (79.0)	6.8 (1.3)
Pneumonia	66 (16.8)	64 (97.0)	7.5 (1.8)	48 (13.0)	46 (95.8)	7.5 (1.9)
COPD exacerbation	26 (6.6)	23 (88.5)	6.4 (1.3)	21 (5.7)	21 (100.0)	7.0 (0.8)
Bronchoaspirative RTI	39 (9.9)	37 (94.9)	6.7 (2.0)	35 (9.5)	32 (91.4)	6.3 (1.8)
Influenza	18 (4.6)	11 (61.1)	6.6 (1.2)	11 (3.0)	11 (100.0)	7.55 (1.2)
COVID-19 infection	7 (1.8)	6 (85.7)	6.3 (1.6)	4 (1.1)	3 (75.0)	5.7 (2.3)
Other RTIs*	26 (6.6)	25 (96.2)	6.7 (1.5)	62 (16.8)	44 (71.0)	6.6 (1.4)
Urinary tract infections	475 (100.0)	446 (93.9)	6.8 (9.3)	297 (100.0)	281 (94.6)	7.2 (16.7)
Cystitis	359 (75.6)	333 (92.8)	7.0 (10.7)	227 (76.4)	216 (95.2)	5.4 (2.6)
Pyelonephritis	12 (2.5)	11 (91.7)	8.3 (3.5)	10 (3.4)	10 (100.0)	8.6 (6.9)
Other UTIs*	78 (16.4)	76 (97.4)	6.4 (2.1)	53 (17.8)	48 (90.6)	7.0 (2.2)

COPD = Chronic obstructive pulmonary disease; RTI = Respiratory tract infection; SD = Standard deviation; UTI = Urinary tract infection

*Corresponding to ‘other’ infections, different from the diagnoses listed above, according to the judgment of the nursing staff professionals

Among residents registered with a suspected urinary tract infection, 6% had an indwelling catheter. The most frequent symptom and sign was acute or worsened confusion, observed in 54.3% of residents, followed by foul-smelling urine and darker urine color, present in 48.6% and 42% of residents, respectively. The most common urinary tract symptom was dysuria, observed in 23.3% of cases, while other symptoms were much less frequent. The presence of common urinary signs and symptoms such as dysuria, urgency, and frequency were significantly more prevalent in the second registration period. Among general signs and symptoms, only fever was more prevalent in the second registration (Table S2). Urine dipsticks were used in 82% of residents with suspected urinary tract infections, and a urine culture was sent to the laboratory in 19% of cases, with no statistical differences between the two registrations.

Cough was the symptom most frequently observed among residents with respiratory tract infections, being present in nearly 70% of cases, followed by increased sputum production and breathlessness, observed in 44.2% and 36.5% of cases, respectively. The presence of cough and sputum volume was significantly more prevalent in the second registration compared to the first audit (Table S3). The most common test performed in these patients was the COVID-19 antigen test, used in 17.9% of cases, while chest X-rays, C-reactive protein rapid tests, and rapid antigen detection tests for streptococcal infection were seldom used. As shown in Table S3, the utilization of tests was not significantly different between the two years, except for the COVID-19 test, which was used more often during the first registration.

## Intervention effect analysis on antibiotic use for urinary and respiratory tract infections and implementation of hygiene elements

Antibiotics were given to 94.2% of the residents with suspected urinary tract infections, with no statistical differences between the two registrations (93.9% in the first registration and 94.4% after the intervention). During the first registration period, 78.1% of residents with respiratory tract infections received antibiotics. However, this rate significantly decreased to 62.7% after the intervention (*P* < 0.001).

Among all the suspected urinary tract infections, potentially unnecessary antibiotic use was 70.3% in the first registration and 59.9% after the intervention, reflecting a significant reduction of 10.4% (*P* < 0.005) (Table 3). In addition, the overall use of first-line antibiotics significantly increased for suspected urinary tract infections in the second registration period, with 17.7% of all prescriptions in the second registration compared to 10.5% in the first audit (*P* < 0.05). The intervention did impact the use of shorter antibiotic courses for cystitis, which dropped from 7.0 days in the first registration to 5.4 days in the second (*P* < 0.05).

**Table 3:** Appropriateness of antibiotic prescription

Characteristics	Total	First registration	Second registration	*P* value
Urinary tract infections, *n* (%)	772	475	297	
Actual antibiotic prescription rate	727 (94.2)	446 (93.9)	281 (94.6)	0.798
Antibiotics theoretically not indicated*	553 (71.6)	361 (76.0)	192 (64.6)	0.001
Potentially inappropriate antibiotic use	512 (66.3)	334 (70.3)	178 (59.9)	0.004
First-line antibiotics given	76 (13.3)	36 (10.5)	40 (17.7)	0.018
Respiratory tract infections**, *n* (%)	649	352	297	
Actual antibiotic prescription rate	449 (69.2)	268 (76.1)	181 (60.9)	< 0.001
Antibiotics theoretically not indicated*	437 (67.3)	239 (67.9)	198 (66.7)	0.803
Potentially inappropriate antibiotic use	255 (39.3)	163 (46.3)	92 (31.0)	< 0.001
First-line antibiotics given	104 (35.6)	56 (32.2)	48 (40.7)	0.173

*Based on the information registered on the form and the current guidelines

**Infections without a specific diagnosis of respiratory tract infection are excluded

Potentially inappropriate antibiotics use for suspected respiratory tract infection was 46.3% in the first registration and dropped to 31% following the intervention (*P* < 0.001) (Table 3). The use of first-line antibiotics for respiratory tract infections increased from 32.2 to 40.7% in the second period, but this change was not statistically significant. The intervention had no impact on the use of shorter antibiotic duration.

A total of 20 nursing homes reported on the implementation of hygiene items across the two registration periods. There was a slight improvement in the number of correctly performed elements in the second registration compared to the baseline results from 2023, albeit without statistical differences. As shown in Table S4, waterproof aprons and eye protection equipment were available in about half of the nursing homes. There was a slight improvement in perineal care performed in bed and the appropriate use of diapers according to the residents’ incontinence after the intervention.

## Discussion

We found a significant reduction in antibiotic prescription rate and in potentially unnecessary antibiotic prescriptions for respiratory and urinary tract infections and in the mean duration of antibiotic courses for uncomplicated urinary tract infections following this intervention, with little effect on the use of first-line antibiotics and number of hygiene elements implemented.

In 2019, the World Health Organization listed antimicrobial resistance as one of the ten threats to global health [20]. To address this threat, antimicrobial stewardship programs, which promote responsible use of antimicrobials, are needed in all settings [21]. However, while these programs are common in hospitals and primary care, they are less prevalent in nursing homes, where antibiotic use is high [22]. Two recent cluster randomized controlled trials conducted in Europe have shown a striking reduction of slightly over 50% in antibiotic prescribing rates in nursing homes in the intervention groups, compared to the control groups, without increasing complications. In the first trial, conducted across four European countries, three of which had low antibiotic prescribing rates, healthcare professionals received a multifaceted stewardship intervention, including a decision tool and educational materials from an expert team. Although initially planned for four months, the intervention was modified due to the COVID-19 outbreak, incorporating education, evaluation, and local adaptation [23]. In a Danish randomized clinical trial, the healthcare professionals in the intervention group attended a 75-min session to learn how to distinguish between UTIs and asymptomatic bacteriuria, evaluate non-specific symptoms, and use a dialogue tool, which was employed when nursing home staff suspected a UTI. This tool included both reflection and communication elements [24]. Two recent systematic reviews of studies on antimicrobial stewardship programs in nursing homes revealed a lack of high-quality interventional studies. However, multifaceted interventions, including education, monitoring, and feedback, appear to be the most promising strategy [25]. The latest meta-analysis found that antimicrobial stewardship interventions were associated with a 14% reduction in antibiotic use. Although study designs varied considerably, the effect size in randomized clinical trials was larger compared to most other studies [26].

Our study showed a reduction in potentially unnecessary antibiotic use among nursing home residents with suspected infections, but the reduction achieved with our intervention was not as relevant as anticipated. When the professional is certain that a resident has a true urinary tract infection, an antibiotic course is justified. However, many suspected infections are not actual infections, as professionals still rely on criteria that are not evidence-based. The presence of asymptomatic bacteriuria is very prevalent among nursing home residents, and the strongest predictors for antibiotic use found in our study, such as acute confusion, foul-smelling urine and dark urine color, are unclear criteria for diagnosing urinary tract infections [27]. Typical urinary signs and symptoms were present in less than a quarter of the suspected urinary tract infections in this study, suggesting a high prevalence of asymptomatic bacteriuria being wrongly treated with antibiotics. However, the intervention had a significant impact, as the prevalence of these symptoms was significantly higher in the second registration compared to the first, suggesting a lower percentage of residents with asymptomatic bacteriuria being treated with antibiotics after the intervention.

Nearly 80% of all respiratory tract infections during the first registration period were treated with antibiotics. There is a growing body of evidence about the self-limiting nature of these infections, even in older people, and in most cases, antibiotic use in this age population causes more harm than benefit [28]. Guidelines clearly recommend against the use of antibiotics for mainly suspected viral infections, such as acute bronchitis, influenza, common cold, and COVID-19, as well as for other common self-limiting infections like rhinosinusitis, otitis, or pharyngotonsillitis, unless a rapid antigen detection test confirms the presence of streptococcal infection, which is uncommon among older people. As also shown in other studies, professionals usually rely upon the presence of some signs and symptoms, such as tonsillar exudate in pharyngotonsillitis or purulent sputum in lower respiratory tract infections [29, 30]. Nursing staff professionals performed better in the second registration period with fewer cases of potentially unnecessary antibiotic use for both respiratory and urinary tract infections, but the reduction in the percentage of antibiotic use in the second registration, despite being statistically significant, was not great, as more than 60% of residents continued receiving antibiotic courses. First-line use of antibiotics slightly improved after the intervention, being significant only for urinary tract infections, as a result of shorter antibiotic courses in cystitis, and not for residents with respiratory infections. Neither did the intervention have any impact on the mean antibiotic duration for respiratory tract infections nor on the hygiene elements implemented.

Growing evidence shows that reasons for potentially inappropriate and first-choice antibiotics are at least partly psychologically and socially rooted, meaning that antibiotic use is as much a behavior as a scientific decision [31]. Changing practice behavior is challenging and requires the implementation of a systematic approach following components of the normalization process theory. This theory suggests that individual and group reflection on the actions needed to reduce inappropriate antibiotic use ensures high-impact and sustainable results [32]. In our study perceived pressure to prescribe antibiotics was associated with antibiotic use for suspected respiratory tract infection, as was observed in other studies [33]. The results of the first registration audit were provided, and professionals were able to check their own practice by comparing these results with the current recommendations based on current guidelines. Our study only considered a punctual intervention, which could explain the limited effect achieved. Multifaceted interventions seem to be more successful than simple interventions, and those that are sustainable over time also achieve better outcomes [25, 34].

The before-and-after design without a control group is a clear limitation. In uncontrolled before-and-after studies, the assumption of causal inference regarding changes observed before and after the intervention is less robust than it would be if a control group were included for comparison [35]. A control group helps provide evidence that changes occurring over time were not due to natural temporal trends or unmeasured events that coincided with the intervention studied. However, the fact that the audit registration was conducted in 2 consecutive years during the same months, with no changes in clinical care across periods, ensures minimal fluctuation in the incidence of infectious diseases. In addition, there was no selection based on severity (as all infectious diseases were required to be registered), and no other interventions took place during this period, which helps minimize the risk of unidentified confounders [36].

One of the main limitations is the dropout rate observed in our study, which exceeded 30% of the baseline nursing homes. However, no significant differences were observed in the general results of the first registration between the initial nursing homes and those that completed both registration periods. Nursing staff participated voluntarily, and volunteers often have a higher interest in quality improvement programs and research compared to the general professional population [37]. The act of participating in this quality control study may have influenced the behavior of the nursing staff. Nevertheless, this Hawthorne effect would affect both the first and second registration periods [38].

Some variables were not included in the forms, which, despite their limitations, contained 88 items. The diagnosis provided by nursing home professionals was based on their clinical judgment, primarily informed by signs, symptoms, and tests performed. While this approach introduces potential bias, it is important to note that this bias likely affected both registration periods similarly. Another limitation is the lack of clinical outcome evaluation, leaving uncertainty about differences in complication rates or clinical failures between groups. However, resident referrals to hospitals were recorded. Since all information was gathered anonymously, correcting incorrect or missing values was not possible. However, completing the forms was straightforward, with nursing staff professionals simply checking off criteria without writing, allowing them to maintain their usual routines during both registration periods.

This pragmatic study, conducted in diverse areas within a country with high antibiotic prescription rates, demonstrates a reduction in potentially unnecessary antibiotics for urinary and respiratory tract infections, as well as a reduction in the duration of antibiotic courses for suspected uncomplicated cystitis. In our study, the intervention included a 2-h workshop where we presented the results from the first registration, facilitated group discussions, and provided an explanation of updated guidelines on antibiotic use, infection management, and infection prevention and control. However, healthcare professionals received the report prior to the workshop, which also served as an initial intervention. The impact of this low-intensity intervention was significant, and although it was not as great as in the two randomized clinical trials, our study was highly pragmatic and much easier to replicate in other areas. There is still room for improvement, highlighting the need for alternative, multifaceted, and sustainable approaches in antimicrobial stewardship strategies in long-term care facilities. Key factors for success may include the active participation of nursing staff and the incorporation of reflection to address the behavioral aspects of decision-making. In addition, a clear emphasis should be placed on two crucial areas. Despite the intervention, the percentage of episodes of asymptomatic bacteriuria and viral respiratory tract infections, such as most upper respiratory tract infections and acute bronchitis, treated with antibiotics remained considerable. Therefore, a more focused intervention is needed, specifically, emphasizing: (1) the importance of typical urinary tract symptoms and debunking myths about the role of general symptoms in urinary tract infections, as well as the limited benefit of urine strips among residents due to the high prevalence of bacteriuria, and (2) the self-limiting nature of most respiratory tract infections and promoting strategies other than antibiotic therapy for managing these conditions.

## Supplementary Information

Below is the link to the electronic supplementary material.

Supplementary file1 (DOCX 694 KB)

## Data Availability

Data for this study will be made available to others in the scientific community upon request after publication.
