# Supplementary material for: The impact of a multifaceted intervention on antibiotic use for common infections in nursing homes in Spain. A before and after study
Source: Eur Geriatr Med. 2025 Apr 22;16(4):1465–73. doi: 10.1007/s41999-025-01193-0 (PMC12378843; doi:10.1007/s41999-025-01193-0)
Supplement: Supplementary file 1 — Supplementary file1 (DOCX 694 KB) [file 41999_2025_1193_MOESM1_ESM.docx]

**SUPPLEMENTARY MATERIAL**

**Supplementary Table 1.** General characteristics of the residents included in the first registration in the 34 nursing homes who initiated the first audit registration and those who completed the whole study.

| Characteristics | All the nursing homes | Those who completed the study | *P* value |
| --- | --- | --- | --- |
| Number of nursing homes | 34 | 23 |  |
| Number of infections / registrations | 1505 | 1003 |  |
| Age, year, mean (SD) | 85.8 (8.4) | 86.4 (8.2) | .069 |
| Age groups, n (%) | | | |
| <70 years | 83 (5.5) | 49 (4.9) | .182 |
| 70-80 years | 264 (17.6) | 146 (14.6) |  |
| 81-90 years | 668 (44.4) | 459 (45.8) |  |
| >90 years | 489 (32.5) | 348 (34.7) |  |
| Gender, n (%) | | | |
| Men | 463 (30.8) | 291 (29.0) | .375 |
| Women | 1041 (69.2) | 711 (71.0) |  |
| Previous duration of symptoms, mean of days (SD) | 2.5 (3.9) | 2.4 (4.2) | .810 |
| Type of infection, n (%) |  |  |  |
| Urinary tract infection | 719 (47.8) | 475 (47.4) | .095 |
| Respiratory tract infection | 533 (35.5) | 392 (39.1) |  |
| Skin infection | 154 (10.2) | 79 (7.9) |  |
| Another infection | 97 (6.5) | 57 (5.7) |  |
| Antibiotic prescribing rate, n (%) | 1328 (88.2) | 886 (88.3) | .992 |
| Indication for antibiotic therapy, n (%) |  |  |  |
| Initiation of treatment | 1246 (82.8) | 819 (81.7) | .498 |
| Prophylaxis | 43 (2.9) | 37 (3.7) | .296 |
| Continuation | 62 (4.1) | 42 (4.2) | 1.000 |
| Unknown | 5 (0.3) | 3 (0.3) | 1.000 |
| Not given | 147 (9.8) | 107 (10.7) | .506 |
| Antibiotic taken during the previous 15 days, n (%) |  |  |  |
| Yes | 259 (17.2) | 169 (16.8) | .857 |
| No | 1212 (80.5) | 812 (81.0) | .831 |
| Unknown | 9 (0.6) | 8 (0.8) | .728 |
| Where the antibiotic course was initiated, n (%) |  |  |  |
| Nursing home | 1173 (77.9) | 783 (78.1) | .980 |
| Hospital | 151 (10.0) | 94 (9.4) | .633 |
| Health care center | 13 (0.9) | 10 (1.0) | .897 |
| Unknown | 2 (0.1) | 1 (0.1) | 1.000 |
| No antibiotic initiated | 121 (8.0) | 90 (9.0) | .452 |
| Duration of the antibiotic course, mean of days (SD) | 6.8 (6.4) | 7.0 (7.5) | .423 |
| Allergy to penicillin, n (%) | 42 (2.8) | 22 (2.2) | .424 |
| Perception of antibiotic demand, n (%) |  |  |  |
| Yes | 700 (46.5) | 395 (39.4) | <.001 |
| No | 747 (49.6) | 559 (55.7) | .003 |
| Unknown | 31 (2.1) | 28 (2.8) | .294 |
| Referral to hospital, n (%) | 191 (12.7) | 128 (12.8) | 1.000 |

SD=Standard deviation.

**Supplementary Table 2.** General characteristics of the residents with urinary tract infections from the nursing homes that completed the two registration audits.

| Characteristics | Total | First registration | Second registration | *P* value |
| --- | --- | --- | --- | --- |
| Number or registrations / infections | 772 | 475 | 297 |  |
| Catheter indwelling residents, n (%) | 46 (6.0) | 30 (6.3) | 16 (5.4) | .708 |
| Mean prior symptom duration, days, n (SD) | 2.3 (3.2) | 2.5 (3.6) | 1.9 (2.5) | .016 |
| General signs and symptoms, n (%) | | | | |
| Fever | 98 (12.7) | 50 (10.5) | 48 (16.2) | .029 |
| Shaking chills | 62 (8.0) | 37 (7.8) | 25 (8.4) | .860 |
| Confusion | 419 (54.3) | 253 (53.3) | 166 (55.9) | .523 |
| Joint and muscle pains | 37 (4.8) | 18 (3.8) | 19 (6.4) | .140 |
| No general symptoms | 273 (35.4) | 165 (34.7) | 108 (36.4) | .702 |
| Urinary tract signs and symptoms, n (%) | | | | |
| Dysuria | 180 (23.3) | 93 (19.6) | 87 (29.3) | .003 |
| Urgency | 57 (7.4) | 25 (5.3) | 32 (10.8) | .007 |
| Frequency | 66 (8.5) | 28 (5.9) | 38 (12.8) | .001 |
| Incontinence | 29 (3.8) | 19 (4.0) | 10 (3.4) | .798 |
| Back or flank pain | 58 (7.5) | 41 (8.6) | 17 (5.7) | .177 |
| Gross hematuria | 56 (7.3) | 39 (8.2) | 17 (5.7) | .249 |
| Foul-smelling urine | 375 (48.6) | 226 (47.6) | 149 (50.2) | .531 |
| Murky urine | 324 (42.0) | 192 (40.4) | 132 (44.4) | .304 |
| None of the above | 178 (23.1) | 112 (23.6) | 66 (22.2) | .728 |
| Diagnosis, n (%) | | | | |
| Cystitis | 586 (75.9) | 359 (75.6) | 227 (76.4) | .855 |
| Pyelonephritis | 22 (2.8) | 12 (2.5) | 10 (3.4) | .645 |
| Other urinary tract infections | 131 (17.0) | 78 (16.4) | 53 (17.8) | .679 |
| Urine testing, n (%) | | | | |
| Urinary dipstick | 633 (82.0) | 398 (83.8) | 235 (79.1) | .122 |
| Urine culture | 147 (19.0) | 84 (17.7) | 63 (21.2) | .263 |
| No testing | 21 (2.7) | 11 (2.3) | 10 (3.4) | .518 |
| Antibiotic treatment, n (%) | 727 (94.2) | 446 (93.9) | 281 (94.6) | .798 |
| Duration of the antibiotic course, days, mean (SD) | 7.0 (12.7) | 6.8 (9.3) | 7.2 (16.7) | .729 |
| Antibiotic given, n (%) | | | | |
| Penicillin V | 0 (0.0) | 0 (0.0) | 0 (0.0) | NA |
| Amoxicillin | 3 (0.4) | 2 (0.4) | 1 (0.3) | 1.000 |
| Amoxicillin and clavulanate | 72 (9.3) | 43 (9.1) | 29 (9.8) | .839 |
| Macrolide or clindamycin | 2 (0.3) | 1 (0.2) | 1 (0.3) | 1.000 |
| Cephalosporin | 166 (21.5) | 95 (20.0) | 71 (23.9) | .232 |
| Fosfomycin | 298 (38.6) | 179 (37.7) | 119 (40.1) | .558 |
| Nitrofurantoin | 59 (7.6) | 38 (8.0) | 21 (7.1) | .739 |
| Trimethoprim + sulfamethoxazole | 27 (3.5) | 16 (3.4) | 11 (3.7) | .964 |
| Quinolone | 83 (10.8) | 54 (11.4) | 29 (9.8) | .561 |
| Another antibiotic | 29 (3.8) | 23 (4.8) | 6 (2.0) | .070 |
| No antibiotics | 45 (5.8) | 29 (6.1) | 16 (5.4) | .798 |

NA=Not available; SD=Standard deviation.

**Supplementary Table 3.** General characteristics of the residents with respiratory tract infections from the nursing homes that completed the two registration audits.

| Characteristics | Total | First registration | Second registration | *P* value |
| --- | --- | --- | --- | --- |
| Number or registrations / infections | 762 | 392 | 370 |  |
| Mean prior symptom duration, days, n (SD) | 2.2 (2.0) | 2.2 (1.9) | 2.14 (2.0) | .741 |
| General signs and symptoms, n (%) | | | | |
| Fever | 173 (22.7) | 90 (23.0) | 83 (22.4) | .931 |
| Shaking chills | 117 (15.4) | 58 (14.8) | 59 (15.9) | .734 |
| Confusion | 155 (20.3) | 85 (21.7) | 70 (18.9) | .391 |
| Joint and muscle pains | 52 (6.8) | 26 (6.6) | 26 (7.0) | .943 |
| No general symptoms | 403 (52.9) | 197 (50.3) | 206 (55.7) | .154 |
| Respiratory tract signs and symptoms, n (%) | | | | |
| Cough | 532 (69.8) | 252 (64.3) | 280 (75.7) | .001 |
| Rhinorrhea | 213 (28.0) | 102 (26.0) | 111 (30.0) | .253 |
| Otorrhea | 5 (0.7) | 2 (0.5) | 3 (0.8) | .948 |
| Odynophagia | 83 (10.9) | 42 (10.7) | 41 (11.1) | .963 |
| Tonsillar exudate | 17 (2.2) | 15 (3.8) | 2 (0.5) | .005 |
| Tender cervical glands | 27 (3.5) | 19 (4.8) | 8 (2.2) | .071 |
| Dyspnea | 278 (36.5) | 143 (36.5) | 135 (36.5) | 1.000 |
| Increase in sputum volume | 337 (44.2) | 157 (40.1) | 180 (48.6) | .021 |
| Purulent sputum | 107 (14.0) | 52 (13.3) | 55 (14.9) | .595 |
| Bronchospasm | 93 (12.2) | 54 (13.8) | 39 (10.5) | .210 |
| None of the above | 31 (4.1) | 15 (3.8) | 16 (4.3) | .870 |
| Diagnosis, n (%) | | | | |
| Common cold | 184 (24.1) | 87 (22.2) | 97 (26.2) | .226 |
| Acute otitis media | 5 (0.7) | 2 (0.5) | 3 (0.8) | .948 |
| Acute sinusitis | 0 (0.0) | 0 (0.0) | 0 (0.0) | NA |
| Acute pharyngitis | 39 (5.1) | 22 (5.6) | 17 (4.6) | .636 |
| Acute tonsillitis | 10 (1.3) | 8 (2.0) | 2 (0.5) | .134 |
| Acute bronchitis | 139 (18.2) | 77 (19.6) | 62 (16.8) | .349 |
| Pneumonia | 114 (15.0) | 66 (16.8) | 48 (13.0) | .164 |
| COPD exacerbation | 47 (6.2) | 26 (6.6) | 21 (5.7) | .691 |
| Bronchoaspirative respiratory tract infection | 74 (9.7) | 39 (9.9) | 35 (9.5) | .916 |
| Influenza, n (%) | 29 (3.8) | 18 (4.6) | 11 (3.0) | .328 |
| COVID-19 infection, n (%) | 11 (1.4) | 7 (1.8) | 4 (1.1) | .609 |
| Other respiratory tract infections, n (%) | 88 (11.5) | 26 (6.6) | 62 (16.8) | <.001 |
| Tests performed, n (%) | | | | |
| Rapid antigen detection test | 4 (0.5) | 2 (0.5) | 2 (0.5) | 1.000 |
| C-reactive protein rapid test | 26 (3.4) | 10 (2.6) | 16 (4.3) | .251 |
| Chest X-ray | 61 (8.0) | 23 (5.9) | 38 (10.3) | .035 |
| COVID-19 test | 136 (17.8) | 95 (24.2) | 41 (11.1) | <.001 |
| No testing | 541 (71.0) | 250 (63.8) | 291 (78.6) | <.001 |
| Antibiotic treatment, n (%) | 538 (70.6) | 306 (78.1) | 232 (62.7) | <.001 |
| Duration of the antibiotic course, days, mean (SD) | 6.8 (1.7) | 6.7 (1.7) | 6.8 (1.6) | 0.538 |
| Antibiotic given, n (%) | | | | |
| Penicillin V | 0 (0.0) | 0 (0.0) | 0 (0.0) | NA |
| Amoxicillin | 26 (3.4) | 10 (2.6) | 16 (4.3) | .251 |
| Amoxicillin and clavulanate | 247 (32.4) | 132 (33.7) | 115 (31.1) | .492 |
| Macrolide or clindamycin | 43 (5.6) | 25 (6.4) | 18 (4.9) | .455 |
| Cephalosporin | 88 (11.5) | 47 (12.0) | 41 (11.1) | .780 |
| Fosfomycin | 9 (1.2) | 8 (2.0) | 1 (0.3) | .054 |
| Nitrofurantoin | 0 (0.0) | 0 (0.0) | 0 (0.0) | NA |
| Trimethoprim + sulfamethoxazole | 4 (0.5) | 4 (1.0) | 0 (0.0) | .148 |
| Quinolone | 99 (13.0) | 62 (15.8) | 37 (10.0) | .023 |
| Another antibiotic | 35 (4.6) | 22 (5.6) | 13 (3.5) | .226 |
| No antibiotics | 224 (29.4) | 86 (21.9) | 138 (37.3) | <.001 |

COPD=Chronic obstructive pulmonary disease; NA=Not available; SD=Standard deviation.

**Supplementary Table 4.** Percentage of hygiene elements implemented in the two registrations in the nursing homes that provided with information.

| Characteristics | Total | First audit | Second audit | *P* value |
| --- | --- | --- | --- | --- |
| Number of nursing homes | 40 | 20 | 20 |  |
| Hand hygiene, n (%) | | | | |
| Hand sanitizer is hanging beside the resident's front door | 12 (30.0) | 5 (25.0) | 7 (35.0) | .730 |
| Hand sanitizer is hanging beside the care center's front door | 3 (7.5) | 1 (5.0) | 2 (10.0) | 1.000 |
| Always wash hands before and after meeting with residents | 39 (97.5) | 19 (95.0) | 20 (100.0) | 1.000 |
| None of the above | 0 (0.0) | 0 (0.0) | 0 (0.0) | NA |
| Hygiene resources at the nursing home, n (%) | | | | |
| Materials for resident hygiene accessible at strategic points | 38 (95.0) | 19 (95.0) | 19 (95.0) | 1.000 |
| Materials for room cleaning accessible at strategic points | 37 (92.5) | 19 (95.0) | 18 (90.0) | 1.000 |
| Availability of clean/disinfected cloths accessible | 28 (70.0) | 14 (70.0) | 14 (70.0) | 1.000 |
| Accessible trash bins | 37 (92.5) | 19 (95.0) | 18 (90.0) | 1.000 |
| Accessible laundry bins for changing bed linens and towels | 32 (80.0) | 17 (85.0) | 15 (75.0) | .693 |
| None of the above | 0 (0.0) | 0 (0.0) | 0 (0.0) | NA |
| Protective equipment, n (%) | | | | |
| Three sizes of gloves are available in the residents' toilets | 39 (97.5) | 20 (100.0) | 19 (95.0) | 1.000 |
| Waterproof front aprons available for washing residents | 22 (55.0) | 10 (50.0) | 12 (60.0) | .751 |
| Availability of masks | 38 (95.0) | 20 (100.0) | 18 (90.0) | .468 |
| Availability of eye protection | 24 (60.0) | 13 (65.0) | 11 (55.0) | .747 |
| None of the above | 0 (0.0) | 0 (0.0) | 0 (0.0) | NA |
| Specific hygiene resources, n (%) | | | | |
| Availability of wet wipes for changing diapers | 20 (50.0) | 10 (50.0) | 10 (50.0) | 1.000 |
| Availability of hygiene materials, e.g. soapy sponges | 40 (100.0) | 20 (100.0) | 20 (100.0) | NA |
| Waterproof aprons and disposable gloves for perineal care | 28 (70.0) | 14 (70.0) | 14 (70.0) | 1.000 |
| None of the above | 0 (0.0) | 0 (0.0) | 0 (0.0) | NA |
| Professional hygiene management, n (%) |  |  |  |  |
| I always wash my hands before and after attending residents | 36 (92.3) | 18 (90.0) | 18 (94.7) | 1.000 |
| I keep my nails short and clean and remove hand jewelry | 30 (76.9) | 14 (70.0) | 16 (84.2) | .501 |
| None of the above, n (%) | 1 (2.6) | 1 (5.0) | 0 (0.0) | 1.000 |
| Resident and family hygiene management, n (%) | | | | |
| Residents are encouraged to wash their hands | 37 (94.9) | 19 (95.0) | 18 (94.7) | 1.000 |
| We educate family members to wash their hands frequently | 22 (56.4) | 10 (50.0) | 12 (63.2) | .613 |
| None of the above | 2 (5.1) | 1 (5.0) | 1 (5.3) | 1.000 |
| Perineal care, n (%) | | | | |
| Perineal care is always performed in bed | 12 (30.8) | 4 (20.0) | 8 (42.1) | .251 |
| Thorough perineal care is performed at least once a day | 38 (97.4) | 20 (100.0) | 18 (94.7) | .979 |
| Used perineal cleaning towels are not reused for other tasks | 30 (76.9) | 15 (75.0) | 15 (78.9) | 1.000 |
| None of the above | 0 (0.0) | 0 (0.0) | 0 (0.0) | NA |
| Diaper changing, n (%) | | | | |
| The smallest suitable diaper for the resident's size is chosen | 15 (38.5) | 8 (40.0) | 7 (36.8) | 1.000 |
| The diaper is chosen according to the resident's incontinence | 19 (79.2) | 8 (66.7) | 11 (91.7) | .315 |
| Diapers are changed at least 3 times a day | 35 (89.7) | 19 (95.0) | 16 (84.2) | .560 |
| Diaper changing is done according to a fixed schedule | 22 (56.4) | 11 (55.0) | 11 (57.9) | 1.000 |
| Diaper changing is individualized for each resident | 31 (79.5) | 16 (80.0) | 15 (78.9) | 1.000 |
| None of the above | 0 (0.0) | 0 (0.0) | 0 (0.0) | NA |

NA=Not available.

**Supplementary Fig. 1.** Registration form used during the two registration periods.


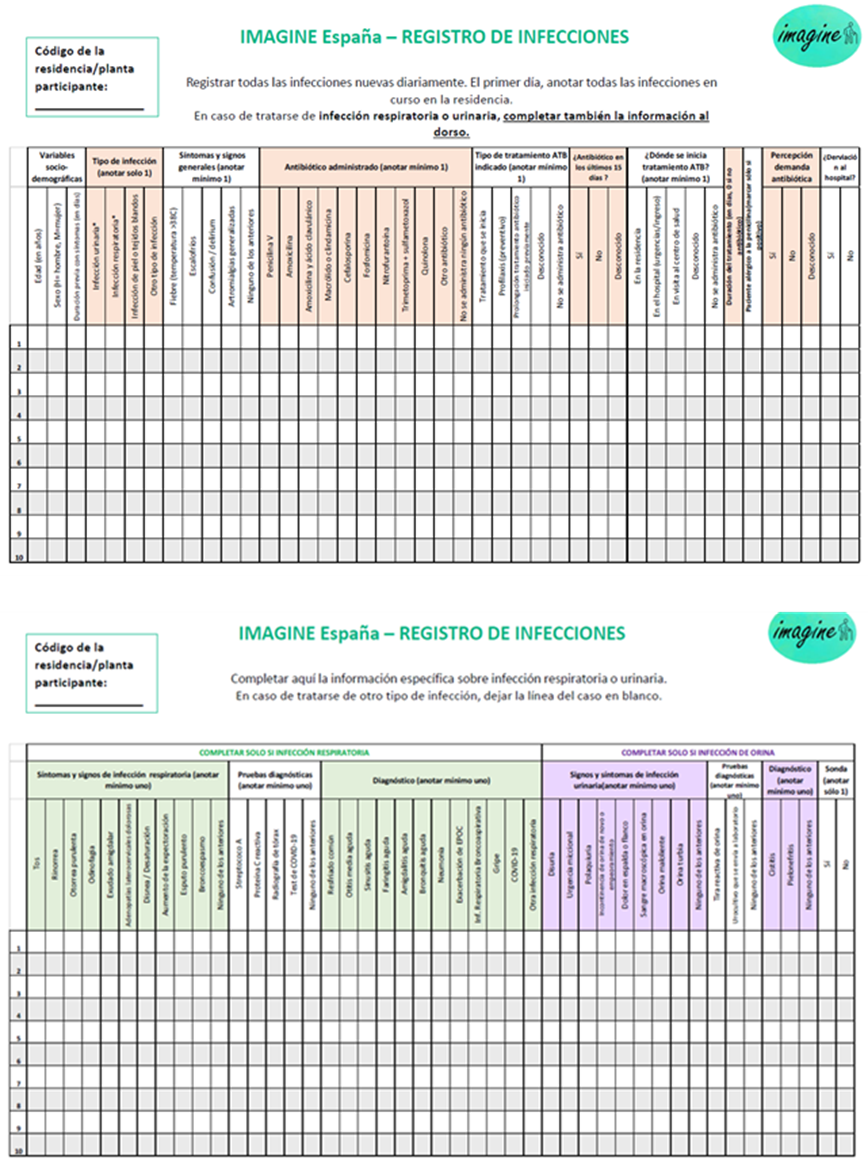


**Supplementary Fig. 2.** Definition of potentially inappropriate antibiotic used in this study.

Appropriateness of antibiotic use

Urinary tract infections

Respiratory tract infections

Antibiotic use is warranted

Potentially inappropriate antibiotic use

Potentially inappropriate antibiotic use

Antibiotic use is warranted

- - Otitis (with otorrhea)
- Amoxicillin 5-7d
  - Sinusitis (with fever, shaking chills or >10 days with symptoms)
- Amoxicillin 5-7d
  - Pharyngitis/Tonsillitis (with StrepA test)
- Penicillin V 7-10d
  - Pneumonia
- Amoxicillin and clavulanate 5-7d
  - COPD exacerbation (with purulent sputum)
- Amoxicillin and clavulanate 5-7d
  - Bronchoaspirative respiratory infection
- Amoxicillin and clavulanate 5-7d
  - <2 lower urinary tract infection signs and symptoms*
  - 1 lower urinary tract infection sign and symptom* or flank pain without (fever/shaking chills OR confusion/poor general status)
  - Cystitis
- Nitrofurantoin 5-7d
- Fosfomycin 1d
  - Pyelonephritis
- Cephalosporin 7d
  - Common cold
  - Otitis (without otorrhea and fever)
  - Sinusitis (without fever, shaking chills or >10 days with symptoms)
  - Pharyngitis/Tonsillitis (without StrepA test)
  - Acute bronchitis
  - COPD exacerbation (without purulent sputum)
  - Influenza
  - COVID

*Dysuria, frequency, urgency, urinary incontinence, suprapubic pain or low abdominal pain, urethral purulence or pain, and/or swelling or tenderness of the testes, epididymis, or prostate. Characteristics of the urine, such as foul-smelling urine, gross hematuria and/or cloudy urine are not included.
